# Influence of the Microalga *Chlorella vulgaris* on the Growth and Metabolic Activity of *Lactobacillus* spp. Bacteria

**DOI:** 10.3390/foods9070959

**Published:** 2020-07-20

**Authors:** Sylwia Ścieszka, Elżbieta Klewicka

**Affiliations:** Institute of Fermentation Technology and Microbiology, Lodz University of Technology, Wólczańska 171/173, 90-924 Łódź, Poland; elzbieta.klewicka@p.lodz.pl

**Keywords:** microalga *Chlorella vulgaris*, lactic acid bacteria, total titratable acidity, enzymatic profile, lactic acid isomers, *Lactobacillus brevis*, algae

## Abstract

The aim of this study was to evaluate the effect of the algae *Chlorella vulgaris* on the growth, acidifying activity, proportion of lactic acid isomers, and enzymatic profile of *Lactobacillus* *brevis* (ŁOCK 0944, ŁOCK 0980, ŁOCK 0992, and MG451814) isolated from vegetable silages. The results indicated that adding algae at concentrations of 0.1% (*w*/*v*) and 1.5% (*w*/*v*) to the *Lactobacillus* spp. growth medium accelerated the growth of bacteria and thus shortened their phase of logarithmic growth. The acidifying activity of the tested *Lactobacillus brevis* increased with an increased concentration of algae. *Lactobacillus* spp. cultured in the presence of *Chlorella vulgaris* showed higher production of l-lactic acid and lower d-lactic acid production. Moreover, the addition of algae changed the enzymatic activity of lactic acid bacteria; for instance, *Lactobacillus brevis* ŁOCK 0980 demonstrated more enzymatic activity of valine arylamidase, α-galactosidase, and α-glucosidase. Combining *Lactobacillus brevis* with the algae *Chlorella vulgaris* allows for the creation of innovative, functional products which confer favorable properties to the final product and open new horizons for the food industry.

## 1. Introduction

Nowadays consumers demand innovative products, and interest in algae has increased significantly. Therefore, several companies have gotten involved in developing foods containing microalgae [1]. Some algae species, such as *Chlorella*, which have great potential for the human diet [2], are already commercially used in foods [3]. Examples of food products with *Chlorella* include organic algae drink and sea-salt-flavored seaweed crackers (Helga brand) produced by company Evasis Edibles (Austria), crispy matcha biscuits by Tohato (Japan), baked bean crackers in the shape of edamame beans from Ginbi (Japan), and greens organic bar (Raw Sun Bite brand) produced by Lavica Food (Poland) [1]. Moreover, several studies have indicated that microalgae can be introduced to food products with high sensorial and nutritional quality; for instance, *Chlorella vulgaris* was incorporated into wheat flour dough and bread [4], breadsticks [5], and cookies [6]. However, only a few studies so far have focused on the practical use of algae and fermented food. Popular fermented products enriched with *Chlorella* are dairy products, such as probiotic fermented milk [7] and various types of cheese (Appenzeller cheese [8] and functional spreadable processed cheese [9]).

The most important microorganisms used in food fermentation are lactic acid bacteria (LAB) [10], while *Lactobacillus* spp. are the utmost studied microorganisms and used as starter cultures [11]. Moreover, 35 *Lactobacillus* species meet the European Food Safety Authority (EFSA) criteria for a qualified presumption of safety (QPS) [12]. Due to their cholesterol-lowering properties [13,14], anti-inflammatory [15], antioxidant [16], and antagonistic activity [17,18,19], LAB are used in the prevention and treatment of many diseases, such as irritable bowel syndrome [20], dysbiosis [18], urinary tract infections [17], antibiotic-associated diarrhea, infectious diarrhea [21], allergies [15], and atopic dermatitis [22,23].

Lactic acid bacteria have also been used for the fermentation of macroalgae (seaweeds) and microalgae, for instance, *Undaria pinnatifida* (Phaeophyta) [24], *Arthrospira platensis* (Spirulina) [25], edible Irish brown seaweeds (*Himanthalia elongata*, *Laminaria digitata*, and *Laminaria saccharina*) [26], and *Chlorella vulgaris* [27].

Both *Chlorella* and *Lactobacillus* fall into the category of Generally Recognized as Safe (GRAS) [28,29], and consuming them offers various health benefits [30,31]. Combining algae with LAB allows for the creation of a functional product with all essential nutrients necessary for the correct and healthy functioning of the body.

The aim of this study was to evaluate the effect of algae *Chlorella vulgaris* on the growth, acidifying (metabolic) activity, and enzymatic profile of four *Lactobacillus brevis* strains.

## 2. Materials and Methods

### 2.1. Bacterial Strains

Four strains belonging to the species *Lactobacillus* brevis which were isolated from vegetable silages were used for the study: *Lactobacillus brevis* ŁOCK 0944, *Lactobacillus brevis* ŁOCK 0980, and *Lactobacillus brevis* ŁOCK 0992—deposited with the Pure Culture Collection of Industrial Microorganisms of the Institute of Fermentation Technology and Microbiology ŁOCK 105, at Lodz University of Technology (Poland)—and *Lactobacillus brevis* MG451814, whose nucleotide sequence is on deposit in the GenBank National Centre for Biotechnology Information database under the accession number MG451814. *Lactobacillus brevis* ŁOCK 0944 has a patent deposit number (B/00035) in the Polish Collection of Microorganisms [32].

Stock cultures of *Lactobacillus brevis* strains were cultured in MRS broth (De Man, Rogosa, and Sharpe, Merck, Germany) at 30 °C for 24 h.

### 2.2. The Research Material

The research material was powdered *Chlorella vulgaris* from Bellis Food (BellisPharma Sp. z o. o., Jarosław, Poland). The algae concentrations used in the study were 0.1% (*w*/*v*) and 1.5% (*w*/*v*).

### 2.3. Statistical Analysis

All experiments were performed in three independent trials. All data are expressed as the arithmetic means of the three repetitions with standard deviations. To determine the statistical significance of differences between the results, the ANOVA test of differences was used, at a significance level of *p <* 0.05. The software Origin Pro 2017 was used for analysis.

### 2.4. Growth of Lactobacillus brevis Cultured in the Presence of Chlorella vulgaris

The effect of algae on the growth of *Lactobacillus brevis* was assessed by incubating the 24-h *Lactobacillus brevis* strains with a density of 10^7^ CFU/mL, in MRS broth (Merck, Germany), in the presence of algae at concentrations of 0.1% (*w*/*v*) and 1.5% (*w*/*v*). The control samples were LAB cultures without algae. The growth of the *Lactobacillus brevis* strains was investigated by the plate method at specified intervals (0, 4, 8, 12, 18, 24, and 48 h) during incubation at 30 °C. After incubation, the cultures were serially diluted, spread on MRS agar plates, and incubated for 48 h at 30 °C. The results are presented as log colony-forming units (CFU) per milliliter.

### 2.5. Acidifying Activity of Lactobacillus spp.

Determination of the total titratable acidity produced by *Lactobacillus brevis* was performed by incubation 24-h *Lactobacillus* strains with 0.1% (*w*/*v*) and 1.5% (*w*/*v*) and without (control samples) *Chlorella vulgaris* and titration samples (after 0, 4, 8, 12, 18, 24, and 48 h of incubation at 30 °C), with a 0.1-N aqueous solution of NaOH, in the presence of phenolphthalein. The total titratable acidity is expressed as mL, and 0.1-M NaOH was used for titration of the 1 mL of sample.

### 2.6. Proportion of Lactic Acid Isomers

The d- and l-isomers of lactic acid produced by the four strains of *Lactobacillus brevis* were evaluated by using the l-/d- Lactic acid assay (Megazyme International Ireland, Bray, Wicklow, Ireland). To check the impact of algae on the chiral purity of the LAB, the bacteria strains were cultured in the presence of *Chlorella vulgaris*, at a concentration of 1.5% (*w*/*v*) for 24 h. The content of l- and d-lactic acid was determined by measuring the increase of NADH at 340 nm.

### 2.7. Measurement of the Enzymatic Profile

The enzymatic profiles of the selected *Lactobacillus* strains were characterized by using the API^®^ ZYM system (BioMérieux, France). To examine the effect of algae on LAB enzymatic activity, *Chlorella vulgaris* was added to the tested samples at a concentration of 1.5% (*w*/*v*). Each well was charged with 65 μL of a LAB cultured with or without algae and incubated at 30 °C for 4 h. After incubation, the reagents ZYM A and ZYM B were added to each well, according to the manufacturer’s instructions, and left for 5 min, to allow the color to develop. Then the strips were placed under a powerful light source for 10 s. The enzymatic activity was evaluated based on the API^®^ ZYM scale provided by the manufacturer, from 0 (no activity) to 5.

## 3. Results

### 3.1. The Impact of Chlorella vulgaris on the Growth of Lactobacillus spp.

**Figure 1:**
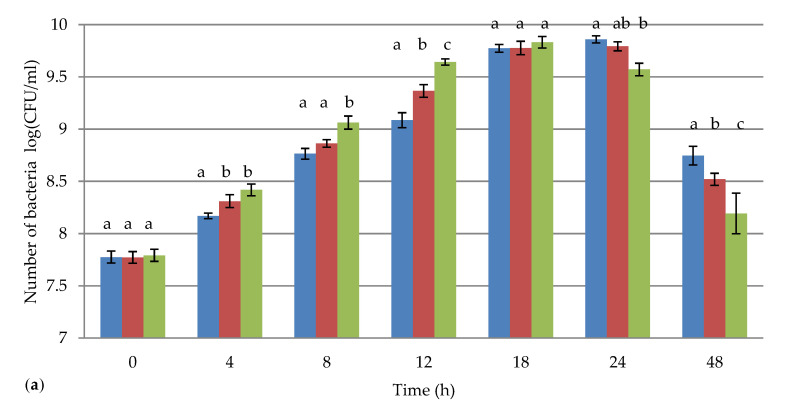

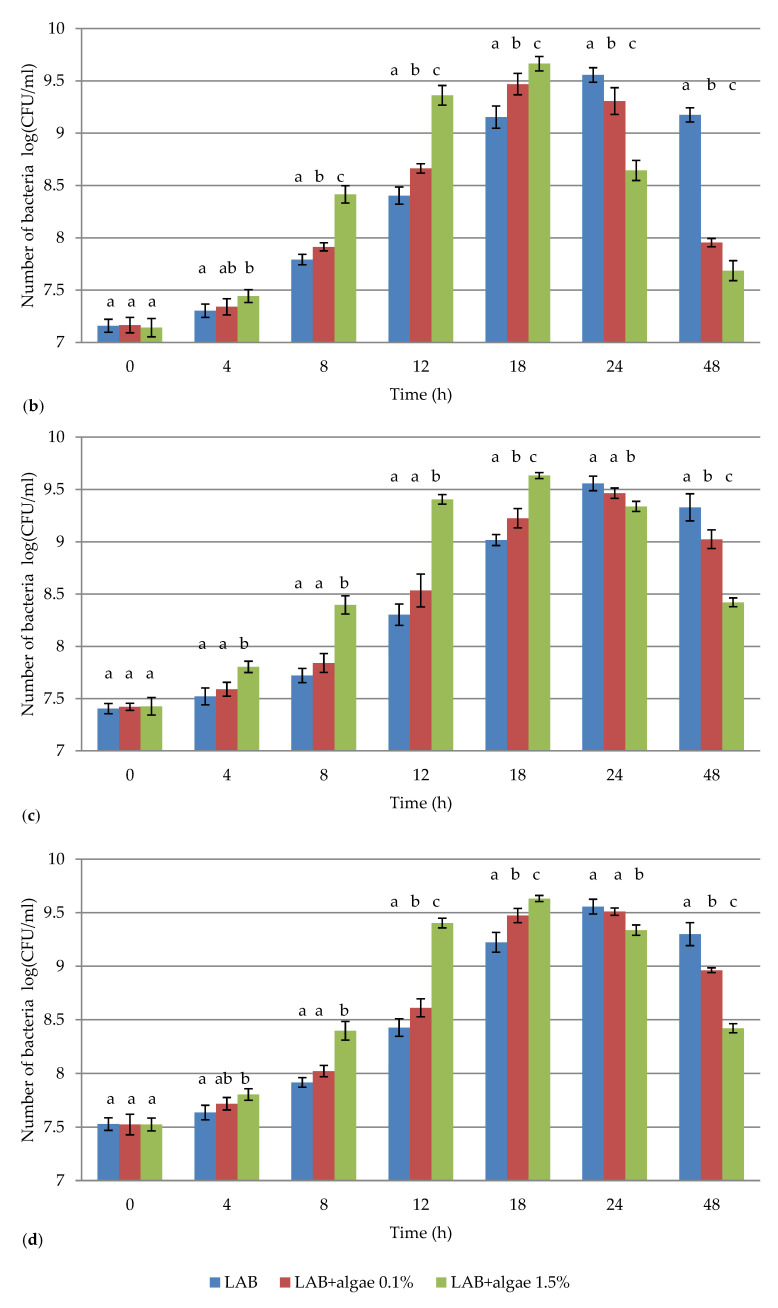
The survival of *Lactobacillus brevis* (**a**) ŁOCK 0944 (**b**) ŁOCK 0980 (**c**) ŁOCK 0992, and (**d**) MG451814 in the presence of the algae *Chlorella vulgaris*, at concentrations of 0.1% (*w*/*v*) and 1.5% (*w*/*v*). Explanatory notes: the figure shows mean values (bars) and standard deviations (line segments); LAB—lactic acid bacteria; a, b, c—the statistically significant differences between samples of the same strains within one time interval; *p <* 0.05; *n* = 3.

The growth of the *Lactobacillus brevis* strains in the presence of *Chlorella vulgaris* at concentrations of 0.1% (*w*/*v*) and 1.5% (*w*/*v*) was tested by the plate method at specified times (0, 4, 8, 12, 18, 24, and 48 h). The results are presented in Figure 1 as the mean values of three independent trials in log CFU/mL.

The results showed that, at concentrations of 0.1% (*w/v*) and 1.5% (*w/v*), *Chlorella vulgaris* had an impact on the number of tested LAB. Statistically significant differences were observed between the control samples and the samples with algae added at a concentration of 1.5% (*w/v*) at all tested hours. The addition of algae at a concentration of 1.5% (*w/v*) had the least impact on the growth of *Lactobacillus brevis* ŁOCK 0944: After 12 h of culturing, the difference between the control sample and the sample with algae was 0.56 log CFU/mL, whereas, in the case of the *Lactobacillus brevis* ŁOCK 0992, the difference in bacterial survival was the largest, at 1.10 log CFU/mL. Moreover, only for *Lactobacillus brevis* ŁOCK 0944 after 18 h of culturing were there no statistically significant differences between all samples.

For all tested *Lactobacillus brevis* strains, the dynamic growth of biomass of the bacteria cultured in the presence of *Chlorella vulgaris* was observed for the first 18 h of culturing. However, for the control samples—which were not enriched with algae—an increase in the number of tested LAB was observed after 24 h of culturing. Therefore, *Chlorella vulgaris* accelerated the growth of *Lactobacillus brevis*, thus shortening their phase of logarithmic growth.

### 3.2. The Effect of Chlorella vulgaris on the Total Titratable Acidity of Lactobacillus spp.

The acidifying activity of the tested *Lactobacillus brevis* strains cultured with and without the algae *Chlorella vulgaris*, at concentrations of 0.1% (*w/v*) and 1.5% (*w/v*), during 48 h of incubation at 30 °C, is presented in Table 1, Table 2, Table 3 and Table 4. The total acidity is expressed as mL 0.1-M NaOH/1 mL of sample.

The results indicated that the acidifying activity of *Lactobacillus brevis* varied depending on the strains of bacteria used. The highest total acidity was obtained for *Lactobacillus brevis* ŁOCK 0944 (2.50 ± 0.05 mL 0.1-M NaOH/1 mL after 48 h of incubation), whereas the lowest was for *Lactobacillus brevis* ŁOCK MG451814 (1.45 ± 0.05 mL 0.1-M NaOH/1 mL after 48 h of incubation).

The acidifying activity of the tested *Lactobacillus brevis* strains increased with an increased concentration of algae. Statistically significant differences between samples with different algae concentrations were visible already at 0 h, because algae acidify the environment. The highest titratable acidity for all tested LAB occurred in the first 24 h of incubation, and the maximum was reached after 48 h. The highest total acidity obtained from a LAB cultured in the presence of *Chlorella vulgaris* was from *Lactobacillus brevis* ŁOCK 0992 (2.70 ± 0.05 mL 0.1-M NaOH/1 mL). Statistically significant differences in total activity between the control samples and the samples with algae within one time interval were observed for almost all tested LAB. Only for *Lactobacillus brevis* ŁOCK 0944 there were no statistically significant differences between the control sample and the sample with algae added at a concentration of 0.1% (*w/v*), after 18 h of incubation.

### 3.3. Influence of Chlorella vulgaris on d- and l-Lactic Acid Production by Lactobacillus spp.

**Figure 2:**
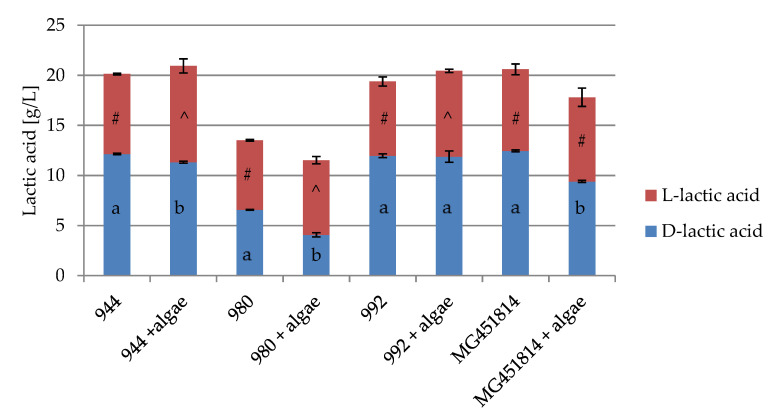
d- and l-lactic acid produced by *Lactobacillus brevis* ŁOCK 0944, 0980, 0992, and MG451814 cultured with and without algae *Chlorella vulgaris.* a, b—the statistically significant differences between samples of the same strains within one time interval for D-lactic acid; *p <* 0.05; #, ^—the statistically significant differences between samples of the same strains within one time interval for l-lactic acid; *p <* 0.05; *n* = 3.

The effect of algae at a concentration of 1.5% (*w/v*) on d-/l- lactic acid production by *Lactobacillus brevis* is presented in Figure 2.

The highest amount of d-lactic acid was found for *Lactobacillus brevis* ŁOCK 0944 (12.15 g/L), while the lowest was *Lactobacillus brevis* ŁOCK 0980 (6.56 g/L). The addition of *Chlorella vulgaris* to the LAB growth medium caused a decrease in the amount of d-lactic acid by all tested *Lactobacillus brevis* strains (statistically significant differences were observed for *Lactobacillus brevis* ŁOCK 0944, ŁOCK 0980, and MG451814). The highest difference in d-lactic acid production was observed by *Lactobacillus brevis* MG451814 (reduction by 3.05 g/L). The l-lactic acid production of the tested LAB strains was similar (6.95–8.15 g/L). *Lactobacillus brevis* cultured in the presence of *Chlorella vulgaris* produced more l-lactic acid (statistically significant differences were observed for *Lactobacillus brevis* ŁOCK 0944, ŁOCK 0980, and ŁOCK 0992). The largest increase (by 1.65 g/L) was recorded for *Lactobacillus brevis* ŁOCK 0944.

### 3.4. The Impact of Chlorella vulgaris on the Enzymatic Activity of Lactobacillus spp.

The enzymatic activity of the *Lactobacillus* strains, which was measured by the API^®^ ZYM, is demonstrated in Table 5. All tested bacteria showed high enzymatic activity (3–5 on the API^®^ ZYM scale) for leucine arylamidase and β-glucosidase. The valine arylamidase activity was high for two strains of *Lactobacillus brevis* (ŁOCK 0980 and MG451814), while, for the other two (*Lactobacillus brevis* ŁOCK 0944 and ŁOCK 0992), the activity for this aminopeptidase was low (2 on the API^®^ ZYM scale). High (5 on the API^®^ ZYM scale) β-galactosidase activity was obtained for *Lactobacillus brevis* ŁOCK 0980 and MG451814, and high acid phosphatase activity was obtained for *Lactobacillus brevis* ŁOCK 0980.

The addition of the algae *Chlorella vulgaris* at a concentration of 1.5% (*w/v*) to a LAB culture caused a decrease or an increase in enzymatic activity, depending on the strain. *Lactobacillus brevis* ŁOCK 0944 revealed lower α-glucosidase and β-glucosidase activity, while *Lactobacillus brevis* MG451814 demonstrated lower valine arylamidase, cystine arylamidase, and acid phosphatase activity. However, *Lactobacillus brevis* ŁOCK 0980 cultured in the presence of *Chlorella vulgaris* showed higher enzymatic activity for valine arylamidase, α-galactosidase, and α-glucosidase, and *Lactobacillus brevis* ŁOCK 0992 for β-glucosidase and *N*-acetyl-β-glucosaminidase. The largest differences in the activity of LAB cultured in the presence of *Chlorella vulgaris* were observed for the α-glucosidase activity of *Lactobacillus brevis* ŁOCK 0980, where the activity increased from 1 to 4 on the API^®^ ZYM scale.

## 4. Discussion

The growth of LAB on conventional organic matrices such as cabbage, carrot, and milk has been measured by several authors [33,34,35]. They reported that the maximum concentrations reached by different species of *Lactobacillus* varied, ranging from 7 to 10 log CFU/mL. This is consistent with the results obtained in this study, where the maximum concentrations of the tested *Lactobacillus brevis* strains were 9.56–9.86 log CFU/mL. These results showed that the number of tested LAB cultured in MRS was observed to increase for up to 24 h of incubation. Similar results were reported by Matejčeková et al. [33], when incubating *Lactobacillus* plantarum at 37 °C. However, according to Bergqvist et al. [34], *Lactobacillus* *pentosus* isolated from cider extract demonstrated a stationary phase after about 9 h; according to Li et al. [36], there was a stationary phase for *Lactobacillus bulgaricus* ATCC 11842 after about 10 h. Moreover, studies by Yoon et al. [35] demonstrated that the viable cell counts of *Lactobacillus casei*, *Lactobacillus plantarum*, and *Lactobacillus* *delbrueckii* reached 8 log CFU/mL after 24 h and increased to 9 log CFU/mL after 48 h of fermentation at 30 °C. Moreover, they reached the conclusion that extending the fermentation beyond 48 h does not result in a significant increase in the viable cell counts of LAB. In contrast, the results of the current study showed a decrease in the number of LAB after 24 h of incubation for all tested strains (especially for *Lactobacillus brevis* ŁOCK 0944).

The effect of algae on the growth of LAB was also examined. A dynamic growth of *Lactobacillus brevis* biomass cultured in the presence of *Chlorella vulgaris* at concentrations of 0.1% (*w/v*) and 1.5% (*w/v*) was observed for the first 18 h of culturing. The shortening of the logarithmic phase of LAB has great technological importance. Niccolai et al. [25] reached the maximum concentration of *Lactobacillus plantarum* ATCC 8014 cultivated with *Arthrospira platensis* (10.6 ± 0.2 log CFU/mL) after 48 h of fermentation. Moreover, *Lactobacillus* bulgaricus ATCC 11842 cells grown in 2% algal carcass media (a by-product of biofuel production) was approximately 7 log CFU/mL; the control medium, MRS broth, provided better nutrient conditions for this LAB [36].

The results indicated that the acidifying activity of the *Lactobacillus brevis* varied depending on the strain of the bacteria. Similar results were obtained by Abbasiliasi et al. [37], who confirmed that the acidifying activity of *Lactobacillus bulgaricus* and *Lactobacillus casei* varied depending on the types and concentrations of bacteria. According to Yoon et al. [35], *Lactobacillus plantarum* and *Lactobacillus delbrueckii* produced significantly more titratable acidity than *Lactobacillus casei.* Moreover, the acidifying activity of the *Lactobacillus brevis* strain tested in this study increased with increasing concentrations of *Chlorella vulgaris*. Statistical analyses indicated that *Chlorella vulgaris* at a concentration of 1.5% (*w/v*) had significant effects (*p* < 0.05) on the titratable acidity of all tested *Lactobacillus brevis* strains. Similar results were reported by Fadaei et al. [38], who found that the addition of *Spirulina platensis* into yogurt caused an increase in titratable acidity during 270 min of fermentation. The highest change in total acidity for all tested LAB occurred in the first 24 h of incubation, and the maximum concentrations were reached after 48 h. Higher total acidity of samples with algae was probably due to the addition of *Chlorella vulgaris*, which stimulated the growth of *Lactobacillus brevis*. Moreover, the acidification of the broth can increase the LAB antagonist activity and lead to inhibition of the growth of pathogenic bacteria [39].

Furthermore, the addition of *Chlorella vulgaris* to the LAB growth medium caused a decrease in the amount of d-lactic acid production and increase the l-lactic acid production by all tested *Lactobacillus brevis* strains. It is worth noting that the metabolic conversion of l-lactic acid in the human body is faster than for d-lactic acid; therefore, l-lactic acid is preferred for use in food [40].

The authors investigated the enzymatic activity of *Lactobacillus brevis*. The results showed that all tested strains demonstrated high enzymatic activity for leucine arylamidase and β-glucosidase. Depending on the strain of *Lactobacillus brevis*, the valine arylamidase and acid phosphatase activities were higher or lower. Only two strains (*Lactobacillus brevis* ŁOCK 0980 and MG451814) demonstrated β-galactosidase activity. According to Mudryk and Podgórska [41], the level of leucine arylamidase activity is a good measure of the proteolytic activity of bacteria, as it is a peptide bond hydrolyzing enzyme. Abouloifa et al. [42] investigated the enzymatic activity of *Lactobacillus brevis*, *Lactobacillus pentosus*, and *Lactobacillus plantarum* isolated from traditional fermenting green olives. They found that all strains had a high enzymatic activity for leucine arylamidase and valine arylamidase, naphthol-AS-bi-phosphohydrolase, β-galactosidase, and β-glucosidase, while the α-glucosidase activity was lower. Moreover, according to Abouloifa et al. [42], *Lactobacillus brevis* presented the highest β-galactosidase activity, a finding which was also confirmed by the results of this work. Furthermore, the high production of β-glucosidase was previously reported in *Lactobacillus* strains isolated from green olives [43].

The addition of *Chlorella vulgaris* to the *Lactobacillus* spp. culture at a concentration of 1.5% (*w/v*) changed their enzymatic activity. The largest differences in the activity of tested LAB cultured in the presence of algae was found for *Lactobacillus brevis* ŁOCK 0980, which showed higher enzymatic activity for valine arylamidase, α-galactosidase, and α-glucosidase. It is worth mentioning that β-glucosidase plays an important role in the bioconversion of oleuropein to hydroxytyrosol, which is a highly desired antioxidant in foods [43]. Moreover, microbial aminopeptidases provide more catalysis with a wide range of applications in the food industry. Aminopeptidase-mediated bioactive peptide synthesis is more preferable because the process is economical and ecofriendly [44]. Aminopeptidases have also been linked to flavor formation [45]. Therefore, the increased valine activity after introducing algae is a beneficial effect.

Considering the high concentration of LAB (9.63–9.83 log CFU/mL) incubated with *Chlorella vulgaris* for 18 h, the higher l-lactic acid production, and the lower d-lactic production, we can conclude that these algae are promising for the development of fermented foods.

## 5. Conclusions

The effect of *Chlorella vulgaris* at concentrations of 0.1% (*w/v*) and 1.5% (*w/v*) on the growth of *Lactobacillus brevis* (ŁOCK 0944, ŁOCK 0980, ŁOCK 0992, and MG451814) was examined. The LAB strains reached the stationary phase after approximately 24 h, though the addition of algae into the *Lactobacillus* spp. growth medium shortened their logarithmic phase (stationary phase after about 18 h), which has great technological importance. The authors indicated that the total acidity of the LAB cultured in the presence of *Chlorella vulgaris* was higher. Moreover, the acidifying activity of *Lactobacillus brevis* increased with increased concentration of algae. The addition of *Chlorella vulgaris* to the LAB growth medium caused increased l-lactic acid production and decreased the amount of d-lactic acid produced by all tested *Lactobacillus brevis* strains. *Chlorella vulgaris* at a concentration of 1.5% (*w/v*) changed the enzymatic activity of the *Lactobacillus* spp. tested (for instance, the higher enzymatic activity for valine arylamidase, α-galactosidase, and α-glucosidase of *Lactobacillus brevis* ŁOCK 0980). The combination of *Chlorella vulgaris* and *Lactobacillus brevis* shows great potential for creating innovative, functional products that can provide a significant amount of lactic acid bacteria to the consumer, in addition to the nutritional properties of algae, thus conferring additional favorable properties to the final food product.

## Figures and Tables

**Table 1:** Effect of algae *Chlorella vulgaris* on the total titratable acidity of *Lactobacillus brevis* ŁOCK 0944.

Time (h)	LAB	LAB+ Algae 0.1%	LAB+ Algae 1.5%
	Total Acidity (mL 0.1-M NaOH/1 mL)		
0	0.70 ± 0.05 ^a^	0.80 ± 0.05 ^b^	0.90 ± 0.05 ^c^
4	0.80 ± 0.05 ^a^	0.90 ± 0.05 ^b^	1.00 ± 0.05 ^c^
8	1.20 ± 0.10 ^a^	1.30 ± 0.10 ^b^	1.45 ± 0.10 ^c^
12	1.80 ± 0.10 ^a^	1.95 ± 0.15 ^b^	2.20 ± 0.10 ^c^
18	2.40 ± 0.05 ^a^	2.45 ± 0.05 ^a^	2.55 ± 0.05 ^b^
24	2.50 ± 0.05 ^a^	2.50 ± 0.05 ^a^	2.60 ± 0.00 ^b^
48	2.50 ± 0.05 ^a^	2.50 ± 0.00 ^a^	2.65 ± 0.05 ^b^

LAB—lactic acid bacteria; ^a, b, c^—the statistically significant differences between samples of the same strains within one time interval; *p <* 0.05; the number of samples (*n* = 3).

**Table 2:** Effect of algae *Chlorella vulgaris* on the total titratable acidity of *Lactobacillus brevis* ŁOCK 0980.

Time (h)	LAB	LAB+ Algae 0.1%	LAB+ Algae 1.5%
	Total Acidity (mL 0.1-M NaOH/1 mL)		
0	0.70 ± 0.00 ^a^	0.70 ± 0.05 ^b^	0.80 ± 0.05 ^c^
4	0.70 ± 0.05 ^a^	0.80 ± 0.05 ^b^	0.90 ± 0.05 ^c^
8	0.90 ± 0.10 ^a^	0.95 ± 0.15 ^b^	1.25 ± 0.10 ^c^
12	1.20 ± 0.10 ^a^	1.30 ± 0.10 ^b^	1.55 ± 0.15 ^c^
18	1.45 ± 0.05 ^a^	1.60 ± 0.05 ^b^	1.85 ± 0.00 ^c^
24	1.60 ± 0.05 ^a^	1.70 ± 0.00 ^b^	1.90 ± 0.05 ^c^
48	1.60 ± 0.00 ^a^	1.70 ± 0.05 ^b^	1.90 ± 0.05 ^c^

LAB—lactic acid bacteria; ^a, b, c^—the statistically significant differences between samples of the same strains within one time interval; *p <* 0.05; the number of samples (*n* = 3).

**Table 3:** Effect of algae *Chlorella vulgaris* on the total titratable acidity of *Lactobacillus brevis* ŁOCK 0992.

Time (h)	LAB	LAB+ Algae 0.1%	LAB+ Algae 1.5%
	Total Acidity (mL 0.1-M NaOH/1 mL)		
0	0.70 ± 0.05 ^a^	0.70 ± 0.05 ^b^	0.85 ± 0.05 ^c^
4	0.75 ± 0.05 ^a^	0.80 ± 0.05 ^b^	1.00 ± 0.10 ^c^
8	0.95 ± 0.10 ^a^	1.05 ± 0.10 ^b^	1.40 ± 0.05 ^c^
12	1.30 ± 0.15 ^a^	1.45 ± 0.10 ^b^	1.80 ± 0.15 ^c^
18	1.70 ± 0.05 ^a^	1.90 ± 0.05 ^b^	2.30 ± 0.05 ^c^
24	2.40 ± 0.05 ^a^	2.55 ± 0.05 ^b^	2.70 ± 0.00 ^c^
48	2.45 ± 0.05 ^a^	2.60 ± 0.05 ^b^	2.70 ± 0.05 ^c^

LAB—lactic acid bacteria; ^a, b, c^—the statistically significant differences between samples of the same strains within one time interval; *p <* 0.05; the number of samples (*n* = 3).

**Table 4:** Effect of algae *Chlorella vulgaris* on the total titratable acidity of *Lactobacillus brevis* ŁOCK MG451814.

Time (h)	LAB	LAB+ Algae 0.1%	LAB+ Algae 1.5%
	Total Acidity (mL 0.1-M NaOH/1 mL)		
0	0.70 ± 0.05 ^a^	0.70 ± 0.05 ^b^	0.90 ± 0.00 ^c^
4	0.75 ± 0.05 ^a^	0.75 ± 0.05 ^b^	0.95 ± 0.05 ^c^
8	0.80 ± 0.05 ^a^	0.90 ± 0.10 ^b^	1.10 ± 0.15 ^c^
12	0.85 ± 0.05 ^a^	1.10 ± 0.10 ^b^	1.30 ± 0.10 ^c^
18	0.90 ± 0.10 ^a^	1.30 ± 0.15 ^b^	1.45 ± 0.00 ^c^
24	1.40 ± 0.05 ^a^	1.60 ± 0.05 ^b^	1.80 ± 0.05 ^c^
48	1.45 ± 0.05 ^a^	1.65 ± 0.05 ^b^	1.80 ± 0.05 ^c^

LAB—lactic acid bacteria; ^a, b, c^—the statistically significant differences between samples of the same strains within one time interval; *p <* 0.05; the number of samples (*n* = 3).

**Table 5:** Enzymatic profiles obtained by API^®^ ZYM for *Lactobacillus* strains cultured with and without the algae *Chlorella vulgaris.*

Enzymes	0944	0944 + Algae	0980	0980 + Algae	0992	0992 + Algae	MG 451814	MG 451814 + Algae
Control	0	0	0	0	0	0	0	0
Alkaline phosphatase	0	0	0	0	0	0	0	0
Esterase (C 4)	0	0	1	1	0	0	1	1
Esterase lipase (C 8)	1	1	1	1	1	1	0	0
Lipase (C 14)	0	0	0	0	0	0	0	0
Leucine arylamidase	3	3	5	5	3	3	5	5
Valine arylamidase	2	2	3	4	2	2	5	4
Cystine arylamidase	0	0	0	0	0	0	1	0
Trypsin	0	0	0	0	0	0	0	0
α-chymotrypsin	0	0	0	0	0	0	0	0
Acid phosphatase	2	2	5	5	1	1	2	1
Naphthol-AS-bi-phosphohydrolase	2	2	2	2	2	2	2	2
α-galactosidase	0	0	1	2	0	0	2	2
β-galactosidase	0	0	5	5	0	1	5	5
β-glucuronidase	0	0	0	0	0	0	0	0
α-glucosidase	1	0	1	4	0	0	2	2
β-glucosidase	4	3	5	5	3	4	5	5
*N*-acetyl-β-glucosaminidase	1	1	0	0	1	2	0	0
α-mannosidase	0	0	0	0	0	0	0	0
α-fucosidase	0	0	0	0	0	0	0	0

The API^®^ ZYM test scale was used for enzyme quantification, with 0 corresponding to a negative reaction/no activity and 5 corresponding to a maximum reaction (3, 4, or 5 being considered positive reactions).
